# Immune cell infiltration and drug response in glioblastoma multiforme: insights from oxidative stress-related genes

**DOI:** 10.1186/s12935-024-03316-2

**Published:** 2024-04-02

**Authors:** Kan Wang, Yifei Xiao, Ruipeng Zheng, Yu Cheng

**Affiliations:** Department of Neurosurgery, The First Affiliated Hospital of Harbin Medical University, Harbin City, 150001 Heilongjiang Province China

**Keywords:** Glioblastoma, ORGs, Prognostic signature, Immune infiltration

## Abstract

**Background:**

GBM, also known as glioblastoma multiforme, is the most prevalent and lethal type of brain cancer. The cell proliferation, invasion, angiogenesis, and treatment of gliomas are significantly influenced by oxidative stress. Nevertheless, the connection between ORGs and GBM remains poorly comprehended. The objective of this research is to investigate the predictive significance of ORGs in GBM and their potential as targets for therapy.

**Methods:**

We identified differentially expressed genes in glioma and ORGs from public databases. A risk model was established using LASSO regression and Cox analysis, and its performance was evaluated with ROC curves. We then performed consistent cluster analysis on the model, examining its correlation with immunity and drug response. Additionally, PCR, WB and IHC were employed to validate key genes within the prognostic model.

**Results:**

9 ORGs (H6PD, BMP2, SPP1, HADHA, SLC25A20, TXNIP, ACTA1, CCND1, EEF1A1) were selected via differential expression analysis, LASSO and Cox analysis, and incorporated into the risk model with high predictive accuracy. Enrichment analyses using GSVA and GSEA focused predominantly on malignancy-associated pathways. Subtype C of GBM had the best prognosis with the lowest risk score. Furthermore, the model exhibited a strong correlation with the infiltration of immune cells and had the capability to pinpoint potential targeted therapeutic medications for GBM. Ultimately, we selected HADHA for in vitro validation. The findings indicated that GBM exhibits a significant upregulation of HADHA. Knockdown of HADHA inhibited glioma cell proliferation and diminished their migration and invasion capacities and influenced the tumor growth in vivo.

**Conclusion:**

The risk model, built upon 9 ORGs and the identification of GBM subtypes, suggests that ORGs have a broad application prospect in the clinical immunotherapy and targeted drug treatment of GBM. HADHA significantly influences the development of gliomas, both in vivo and in vitro.

**Supplementary Information:**

The online version contains supplementary material available at 10.1186/s12935-024-03316-2.

## Background

Glioblastoma, also termed glioblastoma multiforme, is a grade IV glioma, distinguished as the most prevalent and malignant intracranial tumor, primarily composed of astrocytes. This formidable malignancy can appear in individuals of any age, with a prevalence of approximately 3–4 fresh instances per 100,000 people each year in, accounting for roughly 12 to 15% of all tumors affecting the brain [1]. Regrettably, the prognosis for glioblastoma patients is bleak, with a high recurrence rate. According to the latest clinical data, individuals with untreated glioblastoma typically survive for approximately six months, whereas patients who undergo a comprehensive treatment approach consisting of surgery, radiotherapy, temozolomide chemotherapy, and electric field therapy experience a median survival period of 20.9 months [2]. Advances in molecular oncology have yielded molecular markers that can more precisely predict glioblastoma outcomes and aid in clinical management.

Oxidative stress signifies a condition where oxidative and antioxidative activities are imbalanced in the body, favoring oxidation. The disparity causes an inflammatory invasion by neutrophils, heightened release of protease, and a buildup of oxidative byproducts, specifically reactive oxygen species (ROS), resulting in both normal and abnormal cellular and tissue reactions. In the cancer setting, increased generation of reactive oxygen species (ROS) obstructs the ability of DNA repair mechanisms, leading to a buildup of DNA harm, including alterations in the DNA bases, connections between strands and within strands, and interactions between DNA and proteins. Additionally, heightened levels of H_2_O_2_ and O^2−^ contribute to enhanced cell proliferation, ultimately fostering the development of tumors. A wide range of reactive oxygen radicals includes oxygen-based radicals like ROS (which consist of O^2−^, OH^−^, and H_2_O_2_) and nitrogen-based radicals known as reactive nitrogen species (RNS, which include NO, CO_2_, and ONOO^−^) [3].

Glioma is closely linked to oxidative stress, mainly because of the brain's high metabolism of oxygen, which renders the nervous system susceptible to damage caused by oxidation. The generation of ROS causes oxidative stress, resulting in harm to DNA, which affects the growth and programmed cell death of glioma cells [4]. Based on experimental findings, like the examination of H_2_O_2_-triggered apoptosis in glioma cells, it is evident that oxidative stress hinders development and triggers cell death through a caspase-3-dependent pathway [5]. Moreover, the involvement of ROS is essential in the invasion and movement of glioma cells, controlling the manifestation of intercellular adhesion protein-1 while increasing the levels of MMP-9 and MMP-13 [6]. Studies have demonstrated that scavengers of ROS can diminish the invasive and migratory abilities of malignant glioma cells [7]. Furthermore, ROS has the ability to promote the production of vascular endothelial growth factor, consequently facilitating the process of glioma angiogenesis [8].

In our study, we employed bioinformatic methods to investigate ORGs in GBM, aiming to establish a prognostic model and gain deeper insights into the interactions between GBM and oxidative stress. This approach provides a foundation for inspiring early diagnosis, improving prognosis, and developing novel therapeutic targets in the treatment of glioblastoma.

## Materials and methods

### Data collection

A total of 169 data pairs for TCGA-Glioblastoma (TCGA-GBM), were obtained from TCGA (The Cancer Genome Atlas) accessible at [https://portal.gdc.cancer.gov]. For the validation cohort, the GEO dataset GSE7696 was downloaded, excluding patient samples lacking survival data (n = 80), along with 241 normal human samples from the GTEx database. Prior to analysis, patient biodata was prepared by normalizing expression profiles to transcripts per kilobase. This normalization and the subsequent analyses were conducted using the R programming language. For mRNA annotation and differentiation purposes, Gencode (version 26) GTF files were sourced from Ensemble, available at [http://asia.ensembl.org]. Additionally, clinical data encompassing gender, age, clinical stage, and survival information were retrieved from the TCGA data portal. Samples with a survival duration of less than 30 days were deemed ineligible for the study. The data was normalized using the R package ‘limma’ and then subjected to variance analysis using the R package ‘Deseq2’.

### Identification of differentially expressed genes (DEGs):

We used the R package ‘limma’ to identify DEGs in the normalized gene expression data of TCGA-GBM and normal brain tissue samples from the GTEx database. The R package ‘VennDiagram’ was used to identify overlapping ORGs between DEGs and genes related to oxidative stress, where expression changes with |LogFC|> 1 and adjusted P < 0.05 were considered significant.

### GSVA enrichment analysis

Genomic Spatial Event Analysis (GSVA) is an advanced, non-parametric, and unsupervised technique designed to evaluate transcriptome-wide genomic enrichment. By generating integrated scores for specified genomes, GSVA facilitates the translation of gene-level alterations into pathway-level changes, thus illuminating the biological functions of the samples. In this investigation, genomes were sourced from the Molecular Signature Database (MSigDB, version 7.0). Utilizing the GSVA algorithm, we conducted a comprehensive scoring for each genome to gauge potential shifts in biological function across different samples.

### GSEA enrichment analysis

The Gene Set Enrichment Analysis (GSEA) is a technique that orders genes according to the variation in expression between two types of samples and employs predefined gene sets to determine whether these sets are disproportionately represented at the top or bottom of the ranked list. In our study, GSEA was executed using the “clusterprofiler” and “enrichplot” R packages. The objective of this analysis was to clarify the possible molecular mechanisms that contribute to differences in prognosis among GBM patients. This was achieved by examining variations in signaling pathways between groups with high and low gene expression levels.

### Construction and identification of oxidative stress-related signatures

In our research, the initial step to identify genes significantly linked to Oxidative Stress within the TCGA dataset involved employing univariate Cox regression analysis focused on ORGs. This method was instrumental in evaluating the predictive efficacy of ORGs through Cox regression analysis. Subsequently, to refine the gene profile associated with oxidative stress, we utilized LASSO and Cox regression analysis, executed via the Glmnet software. This approach facilitated the identification of overlapping genes.

For practical application, all TCGA and GEO glioma patients were arbitrarily divided into two cohorts: a training group (n = 120) and a test group (n = 119). The training group calculated prognostic risk scores related to oxidative stress and divided the patient population into two categories based on the median risk score. Kaplan-Meier analysis (KMA) was utilized to compare the overall survival time between the two groups, a method that enabled the comparison of OS times across the groups.

Further, DEG-based Principal Component Analysis (PCA) was conducted employing a statistical software package to assess the multidimensional nature of the data. The performance of the test group regarding oxidative stress for each DEG was normalized, ensuring the validity of the model. The test group served as a crucial component for model validation.

A specific formula was used to calculate the risk score for each individual patient based on the levels of different genes. The risk score is calculated using the following formula: Risk Score = (0.455 × expression level of H6PD) − (0.252 × expression level of BMP2) + (0.161 × expressionlevel of SPP1) − (0.908 × expression level of HADHA) + (0.401 × expression level of SLC25A20) + (0.332 × expression level of TXNIP) + (0.587 × expression level of ACTA1) − (0.253 × expression level of CCND1) − (0.451 × expression level of EEF1A1). This formula encapsulates a comprehensive approach to determining the risk associated with oxidative stress in glioma patients.

### Consensus clustering analysis of differentially expressed ORGs

The R package ‘Consensus Clusterplus’ was utilized to conduct unsupervised consensus clustering analysis, aiming to determine the stability and number of clusters. It employs consensus clustering by k-means method to find patterns associated with oxidative stress differential gene expression. To ensure that our classification was accurate, we performed 1000 replications to categorize patients into various molecular subtypes based on oxidative stress-related differential genes. We next applied KMA to calculate survival differences between parents.

### Correlation of signatures, genotypes and TME associated with ORGs

To assess the percentage of immune cells in 23 glioma immune cell subpopulations, we employed CIBERSORT. We analyzed the level of inflammation in the tumor microenvironment (TME) of glioma patients by employing the single-sample gene set enrichment analysis algorithm. The “ESTIMATE” software assessed immune and tumor purity grades.

### Single nucleotide polymorphism (SNP) and copy number variation (CNV) analysis of mutations

Glioma SNP data was analyzed using Map Toolkit to create a waterfall plot using the ten key genes from Sect. 2.12 depicting mutations in the top 20 genes to explore changes in SNP expression between the two groups. The specific analysis procedure was as follows: the CNV files from the database were first tagged and imported into GenePattern software for CNV analysis, after which the data were visualized using Map Toolkit.

### Creation and validation of nomograms and scoring systems

Prediction curve plots were created using “rms” software. Recipient Operating Characteristics (ROC) curves were used to evaluate the histograms over time.

### Drug sensitivity analysis

The pRRophetic algorithm was employed to forecast drug half-maximal inhibitory concentrations (IC_50_) using a correlated ridge regression model. The model used the TCGA cohort as evaluation data for expression profiles and the Genomics of Drug Sensitivity in Cancer (GDSC) cell line as training data (https://www.cancerrxgene.org/). By analyzing the correlation between mRNA expression and the IC_50_ values of both cisplatin and other commonly used drugs in the TCGA dataset, this study predicted the IC_50_ values using spearman correlation.

### Cell culture

Procell Life Science & Technology (Wuhan, China) provided U251, LN229, U87, and NHA cells, which were cultured at 37 °C in DMEM, (HyClone, USA) supplemented with 10% FBS (Invitrogen, USA) and 5% CO_2_. For the experimental study, cells in the logarithmic growth phase were chosen.

### Clinical sample size collection

We acquired samples of normal brain tissue from 10 patients diagnosed with gliomas between 2020 and 2023 at the Fourth Hospital of Harbin Medical University. Furthermore, we gathered specimens from 10 patients who underwent surgical treatment following severe craniocerebral trauma. The human tissues or specimens used in this study were obtained from previous medical records and data, not specifically collected for this study, and were exempted from informed consent in accordance with national medical ethical standards. The tissues or specimens were used only for this study and not for other purposes, and the excess tissues or specimens will be returned at the end of the study, and no personal information about the source of the tissues or specimens will be disclosed as a result of the study. The study was approved by the Medical Ethics Committee of The Fourth Hospital of Harbin Medical University.

### Cell transfection

The lentivirus shRNA-HADHA was procured from Genechem(Shanghai, China). The sequences for this study are as follows: HADHA-RNAi: 5′-CCTGGTGACAAGATTTGTGAA-3′, and NC-RNAi: 5′-TTCTCCGAACGTGTCACGT-3′. Cells were cultured in 6-well dishes with a concentration of 3–5 × 10^4^ cells/ml and kept at 37 °C for 16–24 h until the cells reached a confluence of 30–50%. The cells were treated with the lentivirus and infection enhancement solution according to the instructions provided by the manufacturer. The medium was replaced 16 h later to facilitate further cultivation.

### Western blot analysis

After the cells were treated according to the procedure, the cells of each group were collected and washed twice with PBS, and then RIPA lysis buffer containing phosphatase inhibitor was added. The lysate was lysed in an ice water bath for 30 min. During lysis, the supernatant was lysed by an ultrasonic cell fragmentation apparatus, and the concentration was detected by the BCA method after centrifugation. The denatured proteins were added to prepared SDS‒PAGE gels at a loading amount of 60 µg per well for electrophoresis separation, and then the proteins in the gels were transferred to PVDF membranes. Initially, the PVDF membranes were soaked in a solution containing 5% skim milk powder for a duration of 2 h. This was then followed by an overnight incubation at a temperature of 4 °C with the primary antibodies. Subsequently, they were washed thrice with 1 × PBST and incubated for 2 h with a secondary antibody (Beyotime, Shanghai, China) at room temperature. After being washed three times with 1 × PBST, the membranes were visualized using a chemiluminescence kit (Beyotime) that enhanced the visibility. Protein bands were analyzed using ImageJ software. GAPDH was applied as a reference, and the primary antibody information is shown in Table 1.

**Table 1:** Specific information about the primary antibody

Primary antibody	Company	Molecular weight	Article no	Dilutions (WB)	Dilutions (IHC)
H6PD	Santa cruz	89 kDa	sc-377180	1:1000	1:200
BMP2	Santa cruz	45 kDa	sc-137087	1:1000	1:100
SPP1	Santa cruz	55 kDa	sc-73631	1:500	1:100
HADHA	Santa cruz	83 kDa	sc-374497	1:1000	1:150
SLC25A20	ABclonal	32 kDa	A23763	1:1000	1:100
TXNIP	Santa cruz	46 kDa	sc-271237	1:1000	1:200
ACTA1	ABclonal	42 kDa	A2319	1:1000	1:100
Cyclin-D1	Santa cruz	37 kDa	sc-8396	1:1500	1:200
EEF1A1	Santa cruz	50 kDa	sc-21758	1:1000	1:100
GAPDH	Santa cruz	37 kDa	sc-47724	1:1000	

### qRT‒PCR analysis

Cells were used to extract total RNA with TRIzol (Invitrogen). Then, the mRNA Reverse Transcription Kit (Roche) was used to synthesize cDNA, following the provided instructions. The SYBR Green RNA Kit (Applied Biosystems, USA) was used to perform quantitative real-time PCR (qRT‒PCR), following the manufacturer’s instructions. The PCR cycling parameters consisted of an initial denaturation step at a temperature of 95  °C for a duration of 30 s, followed by 45 cycles of denaturation at 95 °C for 10 s and annealing at 60 °C for 30 s. The specific sequences of the primers can be found in Table 2. The 2^−ΔΔ^Cq method [9] was utilized to quantify the levels of mRNA expression, with GAPDH acting as the reference gene.

**Table 2:** Primers and probes used for qRT-PCR

Gene	Sequences
H6PD	F: 5′- ACCCAGGCATGTGGAATATG -3′
	R: 5′- GTTGCTCCCAGCAGGATTAT -3′
BMP2	F: 5′- AGACCTGTATCGCAGGCACT -3′
	R: 5′- GTTTTCCCACTCGTTTCTGG -3′
SPP1	F: 5′- CGAGGTGATAGTGTGGTTTATGG -3′
	R: 5′- GCACCATTCAACTCCTCGCTTTC -3′
HADHA	F: 5′- AGGGCTTCCTAGGTCGTAAA -3′
	R: 5′- GCAGCTTCAGACTCGCTAAA -3′
SLC25A20	F: 5′- CTGGGATGTTATCTGGCGTATT -3′
	R: 5′- GGTACCAGTGTACTTGCTTTCT -3′
TXNIP	F: 5′- CCTTCGGGTTCAGAAGATCAG -3′
	R: 5′- GGATCCAGGAACGCTAACATAG -3′
ACTA1	F: 5′- GAGGTATCCTGACCCTGAAGTA -3′
	R: 5′- AAGCTCGTTGTAGAAGGTGTG -3′
Cyclin-D1	F: 5′- AGGCGGAGGAGAACAAACAGA -3′
	R: 5′- GGAGGGCGGATTGGAAATGAA -3′
EEF1A1	F: 5′- TCATTGATGCCCCAGGACAC -3′
	R: 5′- TAGGATGCAGTCCAGAGCCT -3′
GAPDH	F: 5′- CTGGGCTACACTGAGCACC -3′
	R: 5′- AAGTGGTCGTTGAGGGCAATG -3′

### Immunohistochemistry

Glioma specimens of varying grades and normal brain tissue underwent formalin fixation, paraffin embedding, and sectioning into 4 µm slices. These sections were dewaxed, rehydrated, and pre-treated with citrate buffer for antigen retrieval, followed by endogenous peroxidase quenching using 3% H_2_O_2_. To block nonspecific antigenic sites, 10% normal goat serum was applied. Primary antibodies, as specified in Table 1, were incubated overnight at 4 °C. This was followed by the application of a secondary antibody (goat anti-rabbit IgG, 1:5000, Proteintech) and staining with diaminobenzidine tetrahydrochloride (DAB) and hematoxylin. IHC images were captured and analyzed using Image J software to quantify protein expression levels.

### Cell viability analysis

Cells were seeded in 96-well plates at a density of 2 × 10^3^ cells per well using DMEM. Following various treatments, they were cultured for 24 and 48 h. Subsequently, each well received 10 μL of CCK8 reagent (Glpbio, California, USA) and was incubated at 37 °C for 1 h. The microplate reader was used to measure the absorbance at 450 nm for each well.

### Colony formation assay

U251 and LN229 cell suspensions were seeded in 6-well plates at a concentration of 1000 cells per well and cultured for a duration of two weeks under different experimental conditions. After the incubation period, the cells were rinsed using PBS, then treated with methanol and finally stained with 0.1% crystal violet. Colonies comprising 50 or more cells were then enumerated using a microscope.

### Wound healing assay

1.5 × 10^5^ cells/ml were added to 6-well plates for cell seeding. Upon reaching 80–90% confluence, a wound was created using a 200 μL pipette tip, followed by rinsing with PBS and incubation in FBS-free DMEM. Cell migration in the wound area was photographed at 24 and 48 h using a microscope. Wound healing was quantified by measuring the reduction in wound length from the original size using ImageJ software.

### Invasion assay

For the invasion test, the chamber inserts were covered with 40 μL of BD Matrigel (Corning, USA) and left to solidify at 37 °C for 1 h. Around 50,000 cells suspended in 500 µL of DMEM without FBS, were added to the top chamber of the insert. Then, the insert was placed in a 24-well plate filled with 750 µL of DMEM supplemented with FBS. Following a 24 h period, the cells that passed through the insert were immobilized using 4% paraformaldehyde, then subjected to staining with 0.05% crystal violet, and finally quantified under a microscope.

### Xenograft nude mouse model

To explore the impact of HADHA on tumor progression in vivo, we used two GBM cell lines with reduced HADHA expression. These cells were cultured and expanded for subsequent experiments. Harvest the tumor cells using trypsinization, neutralize the trypsin, and then count the cells using a hemocytometer or an automated cell counter. Adjust the cell concentration to the desired density using PBS. We then implanted these modified cells subcutaneously into 4 week-old BALB/c nude mice. Inject the prepared tumor cells subcutaneously into the right underarm of the nude mice using a sterile syringe and needle. Each mouse received an injection of 120 µL containing 2 × 10^7^ exponentially growing cells. Monitor the mice regularly for tumor growth, general health, and any signs of distress. Measure the tumors with calipers and record the dimensions to calculate the tumor volume. Provide appropriate care and nutrition to the mice throughout the experiment.

The formula used to calculate tumor volume is V = (length/2) × width^2^. After one month of implantation, the mice were euthanized, and the tumors were removed, weighed, and captured in photographs for subsequent analysis. All experiments involving animals were carried out in accordance with a protocol that was approved by the Institutional Animal Care and Use Committee of The First Affiliated Hospital of Harbin Medical University.

### Statistical analysis:

We assessed the independent predictive capability of the proposed model by employing a Cox regression model. With R4.1.0, all statistical calculations were carried out. Significant differences between different groups are indicated *p < 0.05, **p < 0.01, ***p < 0.001, ****p < 0.0001. The cutoff for statistical significance was P < 0.05.

## Result

### Identification of DEGs associated with GBM patients and their associated functional enrichment analysis compared to normal tissues

**Fig. 1:**
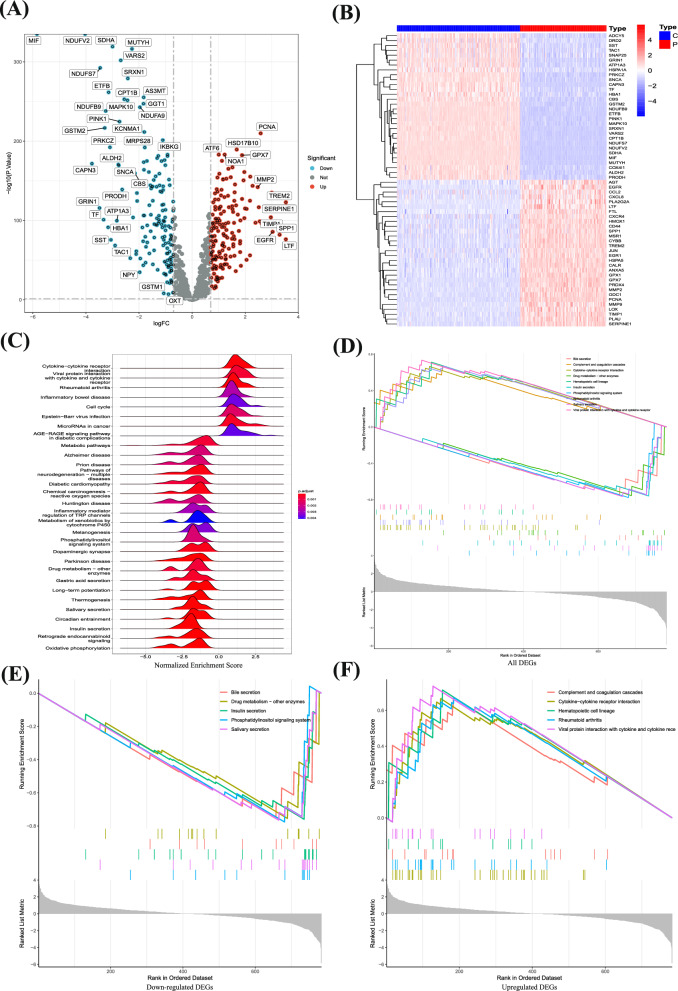
Identification of DEGs and functional enrichment analysis. **A** Screening for DEGs based on the TCGA, GEO combined GTEx cohort. **B** The expression of DEGs in the control group and glioma patients. **C** Enrichment analysis of GSEA in the DEGs. **D**-**F** Enrichment analysis of upregulated and downregulated genes

The analytical process of this study is detailed in Additional file 1: Fig. S1. To delineate the transcriptome of GBM patients, we conducted a screening for differentially expressed genes, focusing on those with a substantial log fold change (|logFC|> 1) and statistical significance (p < 0.05) (Fig. 1A) (Additional file 1: Table S1). This analysis revealed that genes such as ADCY5, DRD2, SST, TAC1, SNAP25, GRIN1, ATP1A3, HSPA1A, PRKCZ, SNCA, CAPN3, TF, HBA1, CBS, GSTM2, NDUFB9, ETFB, PINK1, MAPK10, SRXN1, VARS2, CPT1B, NDUFS7, NDUFV2, SDHA, MIF, MUTYH, COX4I1, ALDH2, and PRODH were underexpressed in tumor tissues, while AGT, EGFR, CCL2, CXCL8, PLA2G2A, LTF, FTL, CXCR4, HMOX1, CD44, SPP1, MSR1, CYBB, TREM2, JUN, EGR1, HSPA5, CALR, ANXA5, GPX1, GPX7, PRDX4, MMP2, ODC1, PCNA, MMP9, LOX, TIMP1, PLAU, and SERPINE1 showed higher expression in tumor tissues (Fig. 1B).

Further exploration into the functions of these GBM-associated differential genes, via GESA, indicated that many of these genes were enriched in pathways related to cytokine receptor interactions, secretions from glands, regulation of coagulation mechanisms, and drug metabolism (Fig. 1C). Additionally, we analyzed the functional aspects of both upregulated and downregulated genes. The findings indicated that the overexpressed genes were primarily enriched in interactions involving receptors for cytokines, lineage of hematopoietic cells, mechanisms of coagulation, rheumatoid arthritis, and interactions between proteins of viruses and receptors of cells. Conversely, downregulated genes were predominantly associated with bile, insulin, and salivary gland secretion, drug metabolism, and phosphatidylinositol signaling systems (Fig. 1D–F). The results indicate that the genes expressed in different ways might have important functions in the growth and advancement of GBM by influencing diverse pathways of communication.

### Determining the association between prognosis and ORGs

**Fig. 2:**
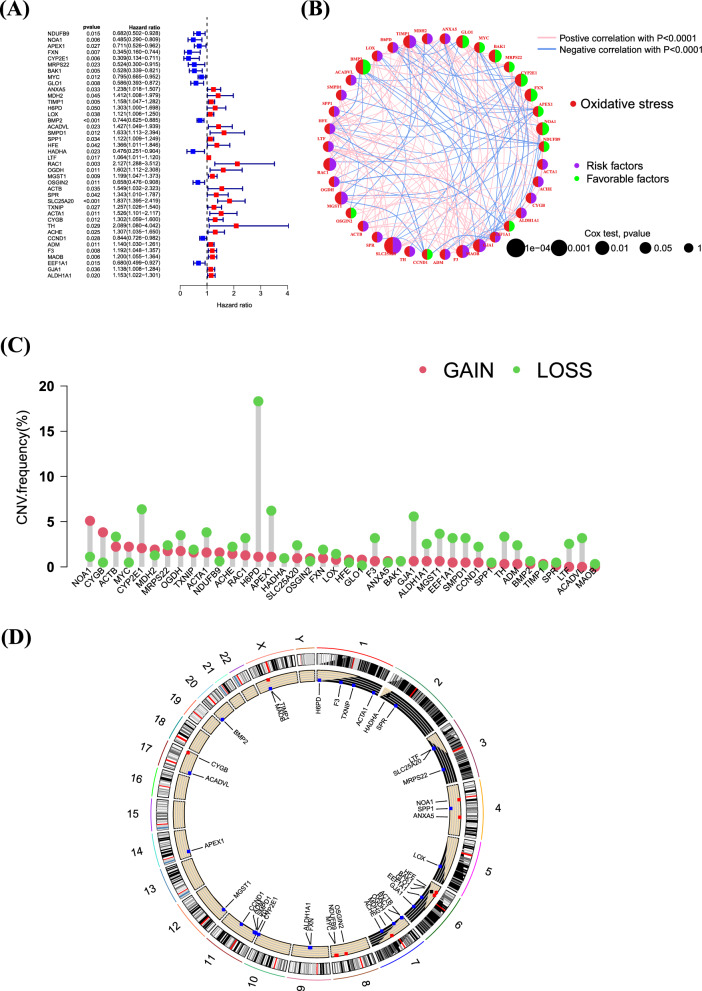
Determining the association between glioma and ORGs. **A** Univariate prognostic analysis of ORGs in glioblastoma. **B** Co-expression interactions between ORGs. **C**, **D** SNP and CNV analysis of mutations in ORGs

To further explore the mechanisms of ORGs in glioma, we merged the survival data of patients and identified 40 ORGs closely related to GBM prognosis through univariate analysis. The analysis results show that these genes are associated with poorer overall survival in GBM patients (Fig. 2A). Through gene prognostic network mapping, we discerned co-expression interactions among these oxidative stress-related differential genes, integral to GBM prognosis. These interactions seemingly play a role in shaping GBM’s formation and progression through mutual regulatory effects (Fig. 2B). Additionally, our study brought to light regulatory mutations associated with oxidative stress, with the most frequent mutation found in H6PD (Fig. 2C–D). Collectively, these findings indicate that the expression levels of ORGs are intricately connected with gliomas, potentially mirroring a spectrum of patient characteristics.

### Consensus clustering identified three relevant isoforms of ORGs

**Fig. 3:**
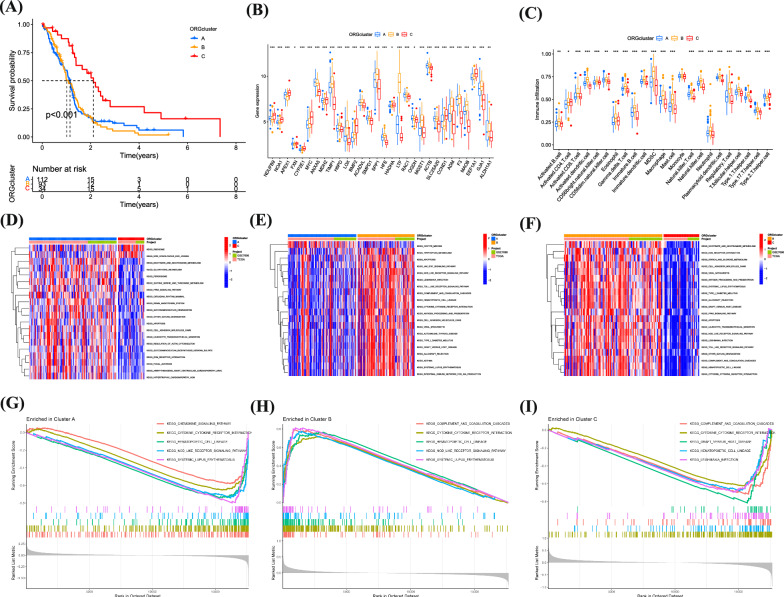
Determining the association between glioma and ORGs. **A** Survival analysis of 3 subgroups. **B**, **C** ssGSEA revealed differential immune cell distributions across the subgroups. **D**–**I** GSEA and GSVA analysis of 3 subgroups

Using expression profiles, we delineated three ORG-associated isoforms for optimal clustering stability at K = 3 (Additional file 1: Fig. S2A). Of the 241 GBM patients studied, 113 were categorized into subtype A, 93 into subtype B, and 35 into subtype C. These ORG-related gene subtypes were sorted into three distinct clusters. Heat maps display the normalized enrichment scores for ORGs across these subtypes (Additional file 1: Fig. S2B–D). Glioma patients were distinctly segregated into all three groups as per PCA and tSNE analyses (Additional file 1:Fig. S2E–G). Survival analysis indicated that each subgroup, demarcated by differences in ORGs, had diverse clinical outcomes. Subtype C patients enjoyed a more favorable prognosis compared to A and B, with B faring the worst (Fig. 3A). We discovered that the expression of these prognostic ORGs varied among the groups. Investigating immune correlations, ssGSEA revealed differential immune cell distributions across the subgroups (Fig. 3B, C). By conducting GSVA analysis pairwise among the three subgroups, the results suggest that in the comparison of the AC subgroups, Ribosome and Non-homologous end joining are significantly enriched in the C subgroup, while Glycolysis, Apoptosis, and PPAR signaling pathway are significantly enriched in the A subgroup (Fig. 3D). In the comparison of the AB subgroups, Oocyte meiosis is significantly enriched in the A subgroup, while Apoptosis, NOD-like receptor signaling, and Tryptophan metabolism are significantly enriched in the B subgroup (Fig. 3E). The BC subgroup comparison analysis shows that ECM-receptor interaction, Starch and sucrose metabolism, and Cell adhesion molecules (cams) are significantly enriched in the C subtype (Fig. 3F). Furthermore, GSEA analysis of the possible mechanisms of action between the three subgroups found that the Chemokine signaling pathway, Cytokine-cytokine receptor interaction, and NOD-like receptor signaling pathway are significantly enriched in the A subgroup. The NOD-like receptor signaling pathway and Complement and coagulation cascades are significantly enriched in the B subgroup, while the C subgroup is significantly enriched in Cytokine-cytokine receptor interaction and Graft versus host disease (Fig. 3G–I). The above results indicate that our model effectively predicts the prognosis of GBM patients and proposes the predictive value of oxidative stress-related gene subtype.

### Modeling oxidative stress-related gene signatures

**Fig. 4:**
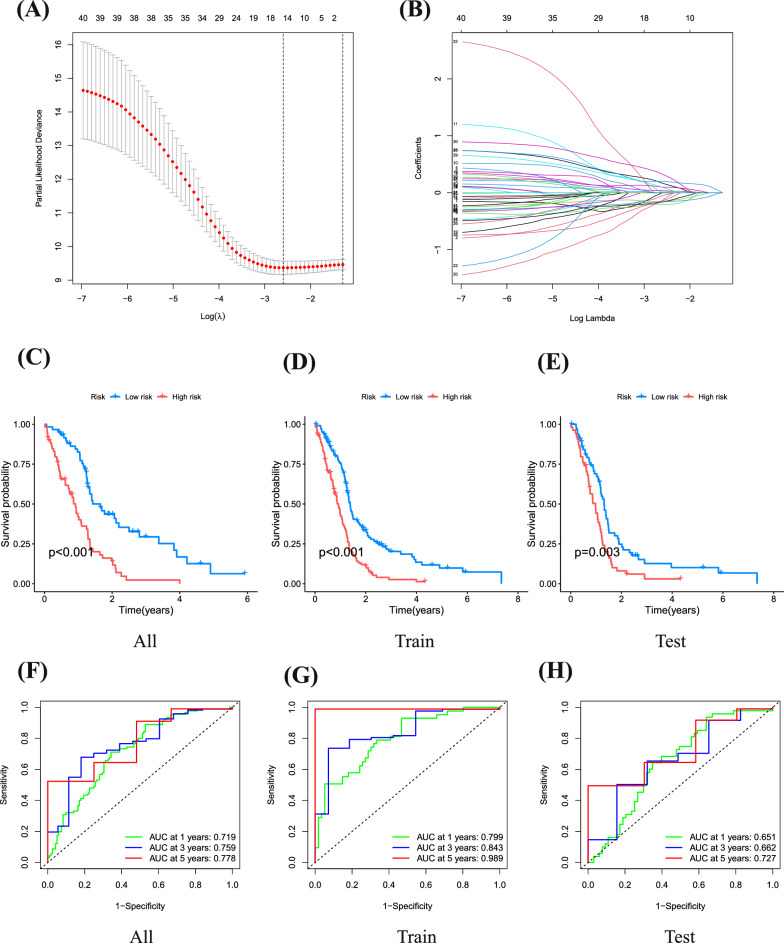
Construction of a prognosis model for glioblastoma associated with ORGs. **A**, **B** LASSO and Cox regression analyses. **C**–**E** Analysis of overall survival in low-risk and high-risk groups. **F**–**H** ROC curves were used to evaluate the histograms over time

We employed LASSO and Cox regression analyses to assess the expression profiles of 40 genes, building a predictive model. Optimal values were used to characterize 9 critical genes. The optimal threshold linked high gene expression with suboptimal outcomes from previous analyses (Fig. 4A, B). Patients were stratified into high-risk (n = 114) and low-risk (n = 125) groups based on the median critical value. High-risk patients, across the overall cohort and in the training and validation groups, exhibited a higher mortality rate than those in the low-risk group, consistently indicating significantly lower overall survival for the high-risk group (Fig. 3C–E). Risk scores were evaluated with Receiver Operating Characteristic (ROC) curves, yielding areas under the curve (AUC) of 0.719, 0.759, and 0.778 for 1, 3, and 5 years, respectively, in the overall cohort. In the training group, AUCs were 0.799, 0.843, and 0.989 for the same time intervals, while the validation group recorded AUCs of 0.651, 0.662, and 0.727 for 1, 3, and 5 years, respectively (Fig. 4F, H). In summary, this paper presents a robust predictive model for risk classification in glioma patients, with consistent validation results across all cohorts, further affirming its sensitivity as a prognostic tool.

### Clinical characterization of different genetic signatures associated with oxidative stress in low and high risk populations

**Fig. 5:**
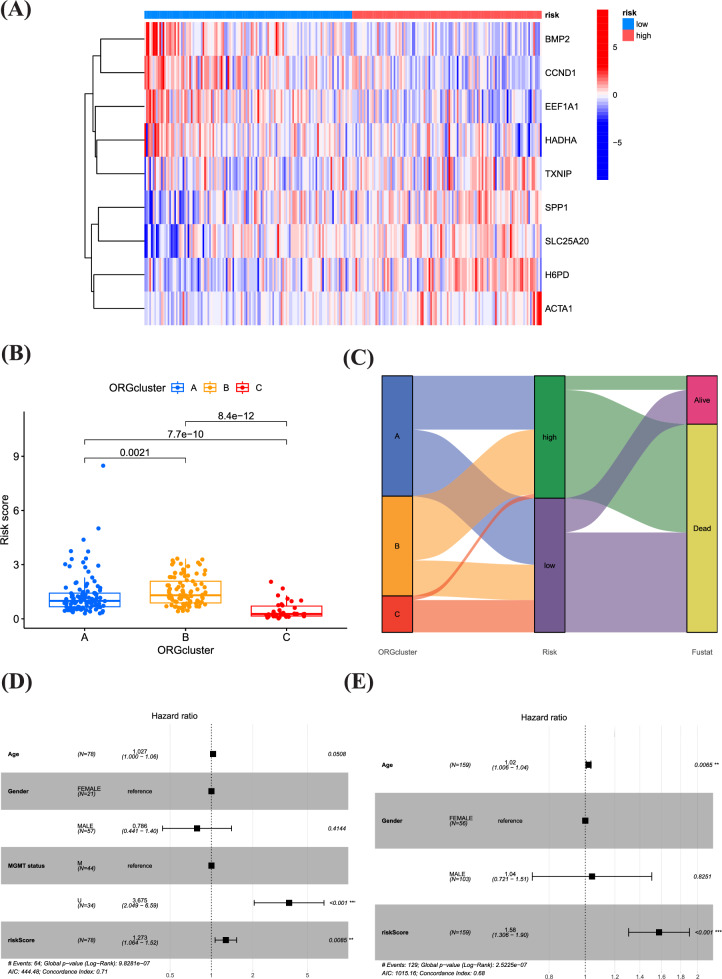
Clinical characterization of different genetic signatures associated with oxidative stress in low and high risk populations. **A** Distribution of ORGs expression in low and high risk populations. **B** Riskscores for three subgroups. **C** Sankey diagram showing the prognosis of four GBM subtypes. **D**, **E** Univariate Cox analysis and Multivariate Cox analysis of the TGGA and GEO cohort

Afterwards, we investigated the possibility of utilizing the oxidative stress pattern to forecast the outcome of GBM individuals, evaluating its practical use in medical environments. Distinct expression patterns were observed in the risk scoring groups. The low-risk group showed predominant expression of BMP2, CCND1, EEF1A1, and HADHA, whereas the high-risk group exhibited notable expression of TXNIP, SPP1, SLC25A20, H6PD, and ACTA1 (Fig. 5A). The mulberry diagram results showed that most patients in group B were placed in the high-risk scoring category, showing lower rates of survival in comparison to group C. Moreover, patients in group B generally displayed higher risk scores than those in groups A and C, which is consistent with our previous analysis findings (Fig. 5B, C). Fig. 5D, E are forest plots of the hazard ratio, showing the effects of age, gender, TMN status, and risk score on outcomes. Our study findings reveal significant associations between the high-risk group and various factors, such as higher tumor grade, older age, and higher risk score. These correlations underscore the relevance of these factors in the context of GBM prognosis and validate the utility of our oxidative stress-based prognostic model.

**Fig. 6:**
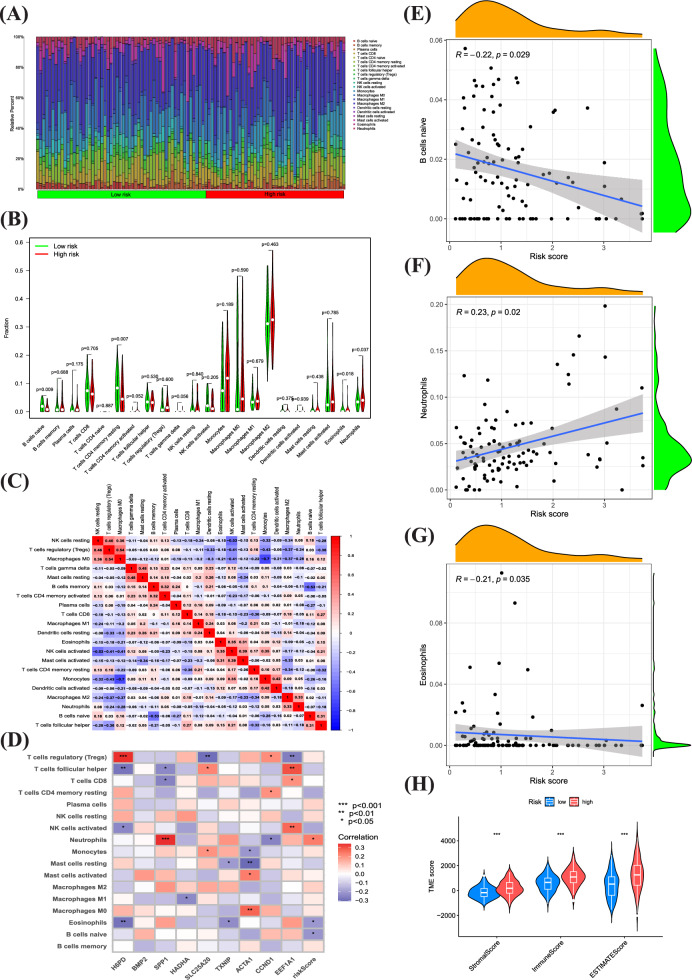
Tumor microenvironmental status in high and low risk scoring groups for oxidative stress. **A**–**C** Correlations between high and low-risk groups and 16 kinds of immune cells. **D** Correlations between ORGs and 16 kinds of immune cells. **E**–**G** Correlations between neutrophils, eosinophils, resident **B** lymphocytes, and risk score. **H** Immune, Stromal, and Estimate Scores in high and low-risk groups

Tumor microenvironmental status in high and low risk scoring groups for oxidative stress Contemporary research indicates that ORGs are crucial in initiating specific anti-tumor immune responses. In this study, we compared the TME compositions of different risk groups. The presence and activity of immune cells in the TME have a profound impact on tumor development and treatment responses. Different types of immune cells, such as T cells, B cells, macrophages, and neutrophils, can be classified based on their roles in anti-tumor or pro-tumor processes. High-risk patients typically had greater proportions of monocytes, M0, M1, and M2 macrophages and patients in the low-risk group have a higher proportion of T cells and NK cells. (Fig. 6A, B). Further, a strong correlation was observed between neutrophils, eosinophils, resident B lymphocytes, and risk score (Fig. 6C–G). Evidence suggests that the altered expression of these genes is key in triggering distinct anti-tumor responses. When examining the TME in groups at different risk levels, we observed differences in ESTIMATE immunological scores. Specifically, the high-risk group had higher scores compared to the low-risk group (Fig. 6H). Based on these findings, we can speculate that variations in the cellular makeup of the TME could play a significant role in the diversity of oxidative stress. Particularly, there seems to be a noteworthy correlation between the extent of immune cell infiltration and the different risk group. Classifying patients with a risk score based on the cellular composition and level of oxidative stress in the TME can help doctors develop more personalized treatment plans for each patient. High-risk patients may require more aggressive treatment methods, including immunotherapy and targeted therapy, while low-risk patients may benefit from more conservative treatment approaches.

### Correlation analysis of risk scores and drug sensitivity analysis

To elucidate the impact of our oxidative stress-related predictive model on GBM medication responses, we delved into the correlation between risk scores and GBM therapeutic drugs. The analysis unveiled a pronounced link between risk scores and drug efficacy. We highlighted certain medications like Bibr-1532, Bi-2536, Niraparib, Venetoclax, BMS-345541, AT13148, UMI-77, Tozasertib, and Dasatinib that displayed significant variations in effectiveness between high-risk and low-risk groups (Additional file 1: Fig. S3). These insights suggest that genes associated with oxidative stress could play a role in GBM treatment resistance and present as potential focal points for GBM drug therapy development.

### Expression level of model genes in target tissues

**Fig. 7:**
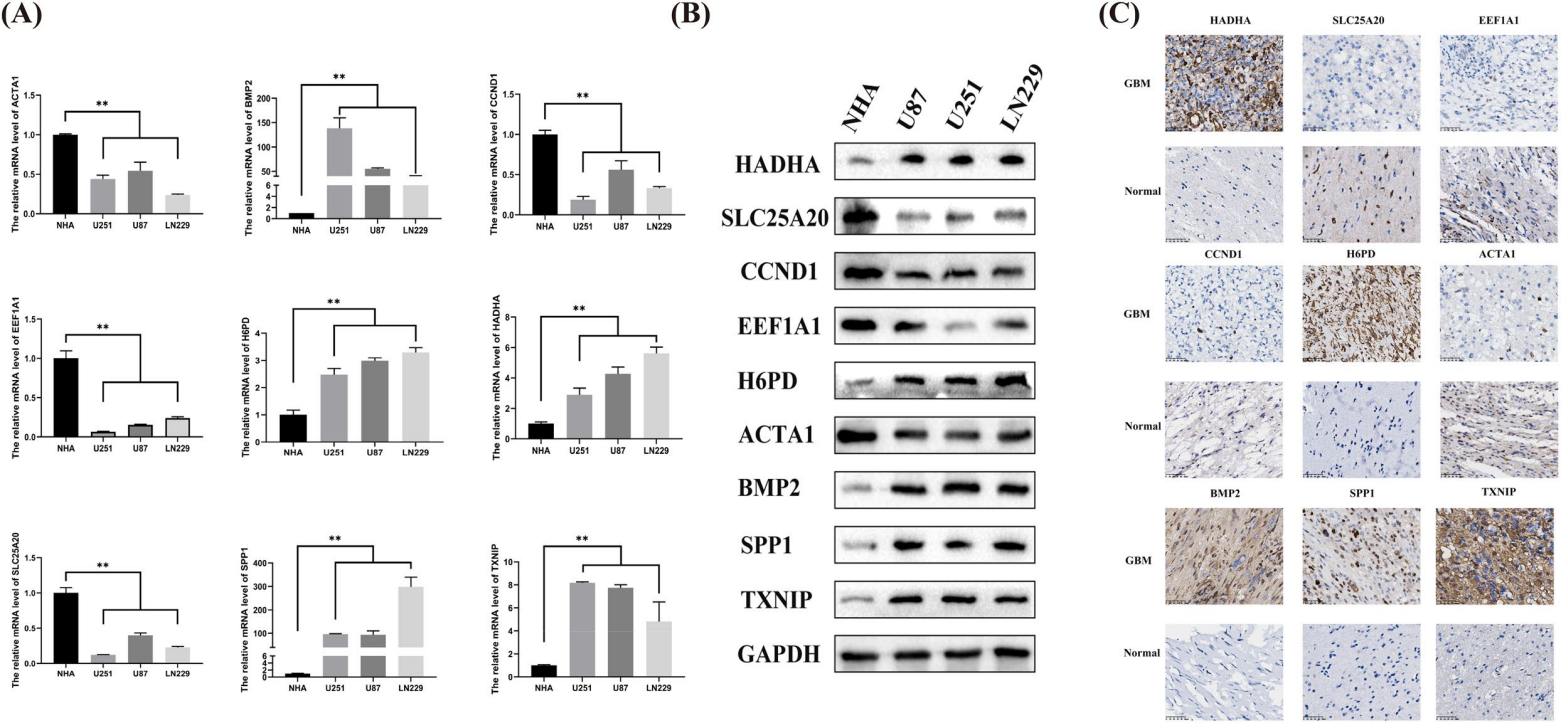
The protein and mRNA expression of ORGs in GBM tissues and cells was detected by IHC, WB and PCR

The qRT-PCR results indicated that the mRNA levels of H6PD, SPP1, SLC25A20, TXNIP, and ACTA1 in GBM cells were considerably greater than those in normal human astrocytes, whereas the expression of BMP2, HADHA, CCND1, and eEF1A1 in GBM cells was notably lower compared to normal human astrocytes. Significantly, the Western Blot test exhibited comparable findings. Furthermore, our immunohistochemical findings additionally demonstrated a substantial upregulation of H6PD, SPP1, and ACTA1 in the GBM patient tissues (Fig. 7). The findings validate that HADHA and BMP2 exhibit significant expression levels in glioma tissues, potentially playing a role in the development and advancement of glioma.

### Knockout of HADHA led to diminished proliferation, migration, and invasion abilities in GBM cells and tumor growth in vivo

**Fig. 8:**
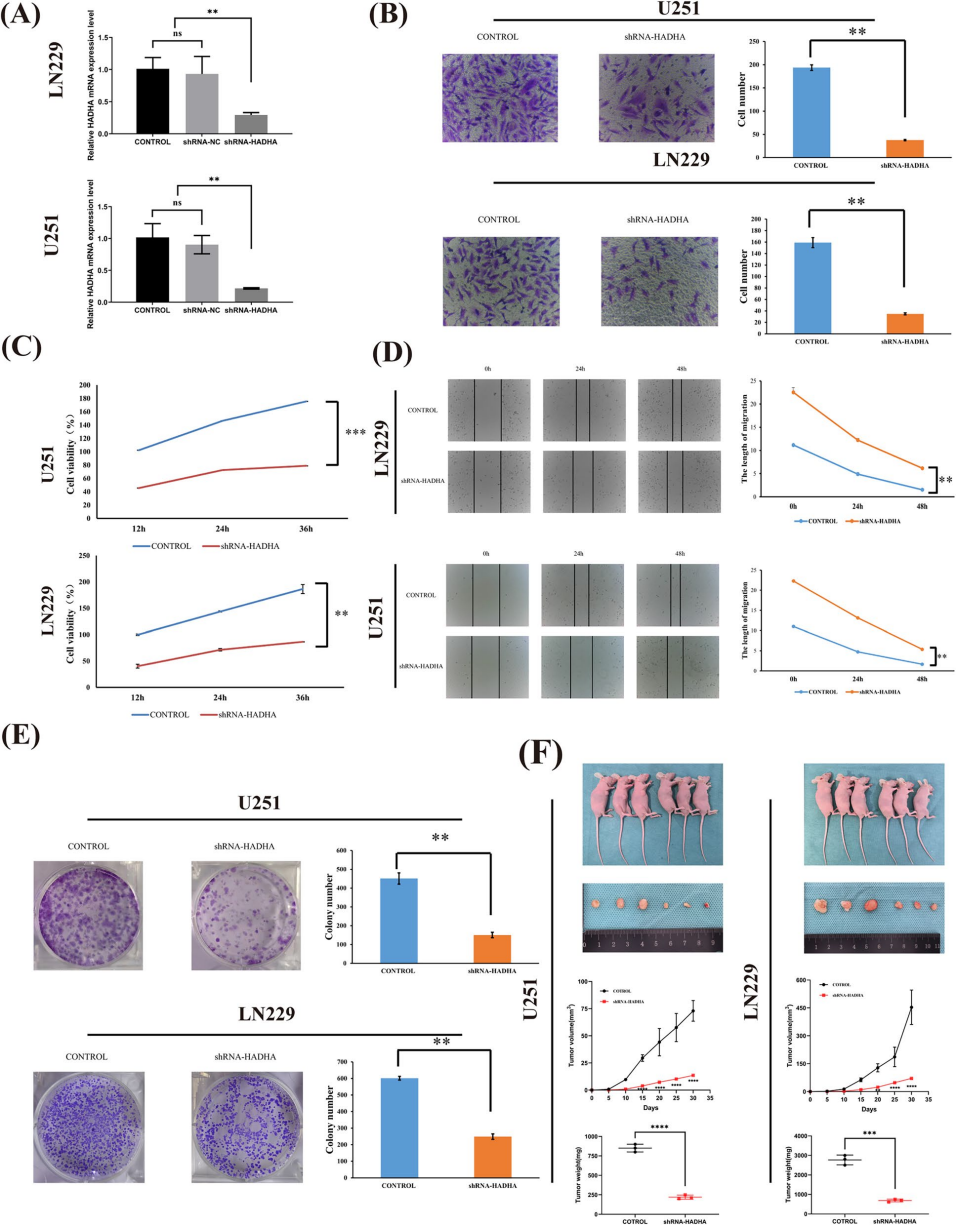
The Impact of HADHA Knockout on GBM cell. **A** After 24 h of transfection, PCR was utilized to analyze the mRNA expression levels of HADHA. **B** Knockdown of HADHA markedly increased the invasion ability of GBM cells. **C** The vitality of transfected GBM cells was measured through CCK8 assay. **D** The migration ability of transfected U251 and LN229 cells was measured through wound healing assay. **E** Knockdown of HADHA significantly reduced the quantity of clones in GBM cells. **F** The knockdown efficiency of HADHA in tumor growth in vivo ** P < 0.01

We transfected HADHA lentivirus into cells to elucidate its role in GBM. To verify the effectiveness of the knockdown, PCR analyses were performed (Fig. 8A). In comparison to the control group, the group with HADHA knockdown showed a notably decreased cell count (Fig. 8B). A series of subsequent experiments revealed significant findings. The CCK8 assay indicated a substantial reduction in cell viability due to HADHA knockdown (Fig. 8C). Wound healing assays showed a marked decrease in the migration ability of U251 and LN229 cells (Fig. 8D). Additionally, HADHA knockdown resulted in a notable decrease in clone cell abundance (Fig. 8E). To deeply assess the effect of HADHA on gliomas in vivo, we chose two cell lines, LN229 and U251, and divided them into control groups and HADHA stable knockdown groups. These cells were then subcutaneously injected into female BALB/c nude mice to establish a xenograft model. As demonstrated in the figure, compared to the control group, the gliomas with HADHA knockdown in both cell lines showed significantly reduced volume and weight (Fig. 8F). Hence, HADHA significantly influences the development of gliomas, both in vivo and in vitro.

## Discussion

GBM represents the most prevalent primary intracranial malignancy in adults. However, even with standard treatment protocols, the median survival duration for GBM patients remains below 14 months [10]. Reactive oxygen species (ROS), products of oxidative stress, are pivotal in influencing the tumor microenvironment of gliomas due to their fluctuating levels. The imbalance of oxidative stress functions as a trigger for the malignant characteristics in clusters of glioma, setting off a sequence of immune-suppressing mechanisms and harmful cellular responses, ultimately leading to the progression of the disease and a grave prognosis [11]. Comprehending the tumor microenvironment affected by ORGs is essential in the creation of a clinical prognosis model, the identification of new markers, and the establishment of risk stratification and therapeutic targets.

In this study, we analyzed ORGs in tumor versus normal tissues. By employing univariate and LASSO regression analyses, we discovered 9 crucial ORGs. Next, a multivariate Cox regression analysis was utilized to calculate coefficients and develop a risk model. According to our research, individuals classified as low-risk experienced extended survival rates in comparison to those categorized as high-risk. To further confirm the accuracy of our model, we created forest and ROC diagrams, in addition to calculating risk scores. The risk model's efficacy was corroborated through risk heatmaps, risk curves, ROC curves, and survival curves, with similar outcomes observed in the validation set.

Within the nine pivotal genes we have discerned, BMP2, HADHA, CCND1, and eEF1A1 emerge as protective factors for the prognosis of GBM, while H6PD, SPP1, SLC25A20, TXNIP, and ACTA1 present as elements of risk. The upregulation of H6PD plays a vital role in the acid-driven purine metabolic reprogramming, conferring a propensity towards the progression of gliomas [12].BMP2 exerts a significant influence on the advancement of gliomas; it can undermine the stability of HIF-1, rendering glioma stem cells more susceptible to temozolomide therapy. Moreover, the expression level of BMP2 is intimately linked to patient survival rates and is considered a prognostic marker for glioma [13].SPP1, a crucial extracellular glycoprotein, is associated with immunomodulation, oncogenesis, and cellular signal transduction [14].Additionally, elevated levels of SPP1 in concert with CD44 correlate with increased macrophage infiltration and an adverse prognosis in patients with neuroglioma [15].TXNIP, a multifaceted protein involved in cellular proliferation, differentiation, and apoptosis, has been shown to promote glioma cell invasion, migration, and proliferation upon downregulation [16].CCND1, a key regulator of the cell cycle, exhibits increased expression with the progression and malignancy of gliomas, portending unfavorable outcomes [17].eEF1A1, a protein ubiquitous in all eukaryotic cells, is essential in the elongation of peptide chains during protein synthesis [18].Its presence is crucial for maintaining the integrity of the cytoskeleton, given its unique binding capabilities with actin, as well as its association with microtubule binding, disassembly, and cell division [19].Literature on SLC25A20, ACTA1, and HADHA remains scant.

By employing the nine predictive genes, we compute a risk assessment for every individual. Patients who are categorized according to the median of these risk scores show a significant disadvantage in terms of survival among those in the high-risk category. Patients in all dataset cohorts, including the training and validation groups, who have higher risk scores, exhibit considerably shorter survival durations in comparison to their low-risk counterparts. Moreover, we observed that the risk score correlates with immune cell expression. As the risk score increases, the quantities of T cells, B lymphocytes, memory cells, NK cells, and T helper cells decrease, whereas the levels of monocytes, M0 macrophages, M1 macrophages, and M2 macrophages rise. The connection implies a suppression of both natural and acquired immune reactions. Despite the increase in macrophages in these circumstances, it could potentially indicate a counteractive response to the inhibition of immune cells. Furthermore, it should be highlighted that tumor-related macrophages have been documented to result in unfavorable consequences in individuals with GBM, suggesting that the immune condition linked to an elevated risk score does, in fact, imply a worse prognosis for GBM patients [20, 21]. Although age and MGMT status affect the overall survival of GBM patients, our risk score significantly improves the prognostic significance of the disease. Immunotherapy has emerged as the fourth major treatment modality for cancer, following surgery, radiotherapy, and chemotherapy. Our risk model, which demonstrates a significant association with tumor immunity and mutation, indicates that immunotherapy could be particularly effective in GBM patients with high-risk scores.

Finally, we have identified several drugs correlated with the risk score. Dasatinib, approved by the FDA for use in GBM, is a central nervous system penetrant [22]. The use of UMI-77, a substance that inhibits Mcl-1, enhances the effectiveness of TRAIL therapy in glioma cells by increasing TRAIL-induced apoptosis. This provides a new approach for treating gliomas [23]. Although it exhibits a tendency to provoke resistance, Tozasertib, a broad-spectrum Aurora kinase blocker, efficiently triggers abnormalities in cytoplasmic division and leads to the demise of high-grade glioma cells in both pediatric and adult [24].

In addition to identifying potential biomarkers in GBM, it is essential to subtype GBM for the improvement of personalized treatment approaches. Based on 34 ORGs, we categorized GBM patients into three GS subtypes. Afterwards, we assessed the prognostic significance, genes specific to each subtype, enriched pathways, and immune infiltration. Our findings reveal that patients within the GS-C subtype have the most favorable prognosis.

Our survival analyses and in vitro studies indicate that four of the nine pivotal genes significantly predict adverse overall survival. GBM tissues exhibit significant upregulation of BMP2, HADHA, CCND1, and eEF1A1 mRNA and protein levels compared to normal tissue. The findings indicate that the proteins produced by these crucial genes might have a potential oncogenic function in GBM. As a result, we selected HADHA for conducting in vitro experiments to determine its impact on the physiological processes of GBM. The results of our study showed that the suppression of HADHA led to a decrease in the proliferation, invasion, and migration of GBM cells, and had an impact on the growth of tumors in vivo. Despite its insights, our study has limitations and necessitates further comprehensive mechanistic research and animal experimentation to investigate the relationship with oxidative stress.

HADHA, central to mitochondrial fatty acid beta-oxidation, plays a significant role in glioma through its impact on energy metabolism, cell proliferation, oxidative stress, immune response, and therapeutic targeting. It influences glioma cell growth and tumor aggressiveness by supporting altered metabolic demands and affecting the balance of reactive oxygen species. This enzyme's activity might modulate the immune microenvironment and response to therapy, making it a potential target for glioma treatment strategies. Understanding HADHA's specific roles could lead to improved therapeutic approaches for glioma patients.

## Conclusion

The present research signifies the first creation and verification of a GBM experiment-derived model for predicting Oxidative Stress. Based on 9 crucial genes, the model acts as a standalone prognostic determinant for patients with GBM. Enhancing precision therapy tailored to patients' clinical profiles and their responsiveness to chemotherapy and radiotherapy could be achieved by our understanding of the Oxidative Stress-based RiskScore for GBM. Furthermore, subsequent studies revealed that HADHA significantly influences the proliferation, invasion, and migration of GBM cells, as well as tumor growth in vivo. In general, these discoveries might encourage additional investigation into focusing on these genes and investigating new mechanistic pathways, potentially leading to the creation of new pharmacotherapies based on gene profiling.

### Supplementary Information

**Additional file 1**: **Figure 1.** The flowchart for the study. **Figure 2.** Consensus clustering analysis of differentially expressed ORGs. A Consensus clustering identified three relevant isoforms of ORGs. B–D Heat maps display the normalized enrichment scores for ORGs across these subtypes. E–G PCA, tSNE and UAMP analyses. **Figure 3.** Analysis of drug sensitivity. A–I Relationship between sensitivity and risk score for nine drugs. **Table S1.** GBM Differential Gene List.

## Data Availability

All data generated or analysed during this study are included in this published article [and its Additional file information files].

## Abbreviations

ORGs: Oxidative stress-related genes
ROS: Reactive oxygen species
GBM: Glioblastoma multiforme
TCGA: The cancer genome atlas
DEGs: Differentially expressed genes
GSVA: Genomic spatial event analysis
GSEA: Gene set enrichment analysis
KMA: Kaplan-meier analysis
PCA: Principal component analysis
TME: Tumor microenvironmental status
SNP: Single nucleotide polymorphism
CNV: Copy number variation
