# Supplementary figures and images for: Immune cell infiltration and drug response in glioblastoma multiforme: insights from oxidative stress-related genes
Source: Cancer Cell Int. 2024 Apr 2;24:123. doi: 10.1186/s12935-024-03316-2 (PMC10986133; doi:10.1186/s12935-024-03316-2)

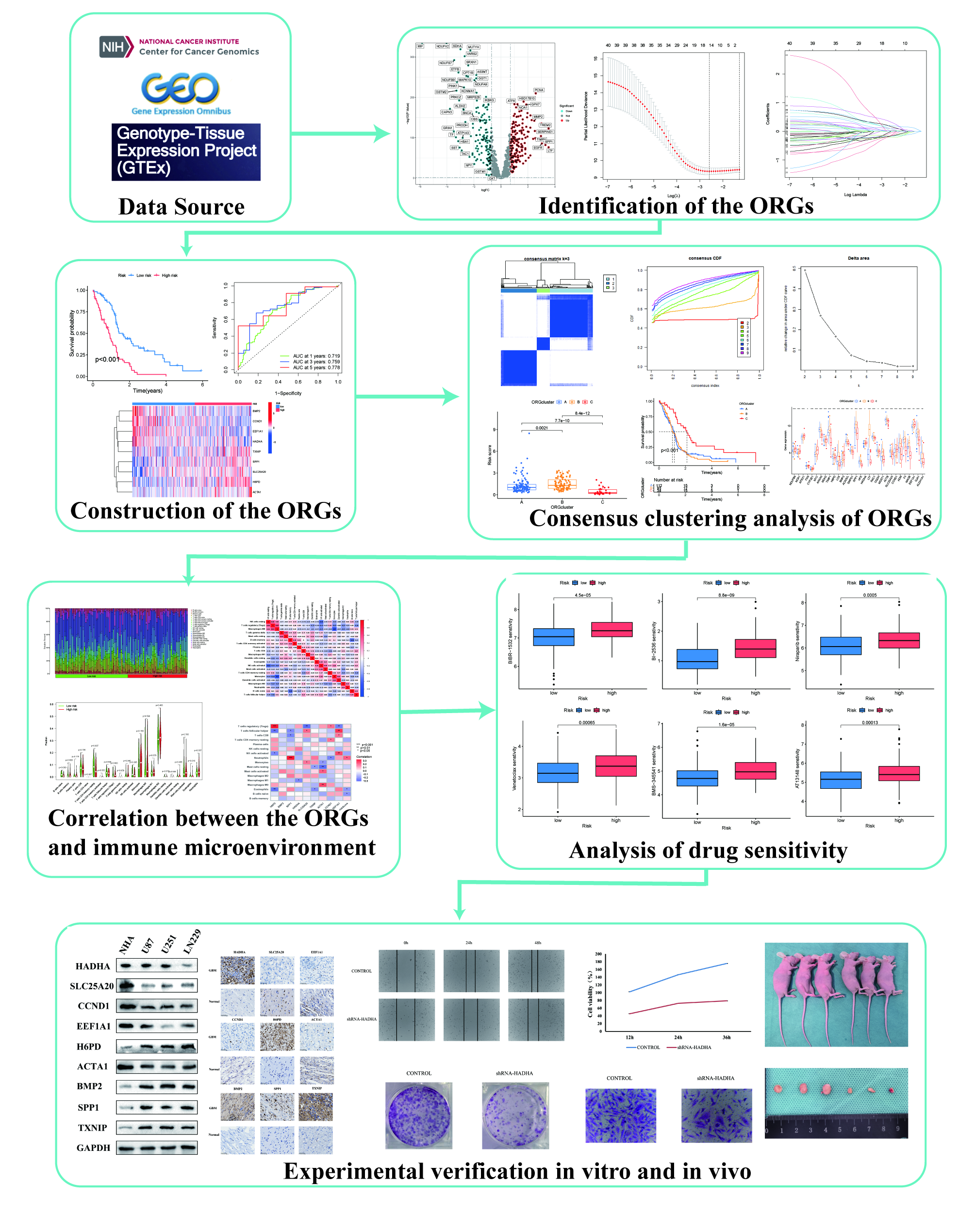

Supplement: Supplementary file 1 — Additional file 1: Figure 1. The flowchart for the study. Figure 2. Consensus clustering analysis of differentially expressed ORGs. A Consensus clustering identified three relevant isoforms of ORGs. B–D Heat maps display the normalized enrichment scores for ORGs across these subtypes. E–G PCA, tSNE and UAMP analyses. Figure 3. Analysis of drug sensitivity. A–I Relationship between sensitivity and risk score for nine drugs. Table S1. GBM Differential Gene List. [file 12935_2024_3316_MOESM1_ESM.zip › Supplementary Figure 1.tif]
